# Prognostic significance of immune landscape in tumour microenvironment of endometrial cancer

**DOI:** 10.1111/jcmm.15408

**Published:** 2020-05-19

**Authors:** Bi‐Lan Li, Xiao‐Ping Wan

**Affiliations:** ^1^ Department of Gynecology Shanghai First Maternity and Infant Hospital Tongji University School of Medicine Shanghai China

**Keywords:** endometrial cancer, immune infiltration, TCGA, tumour microenvironment

## Abstract

Tumour microenvironment (TME) is crucial to tumorigenesis. This study aimed to uncover the differences in immune phenotypes of TME in endometrial cancer (EC) using Uterine Corpus Endometrial Carcinoma (UCEC) cohort and explore the prognostic significance. We employed GVSA enrichment analysis to cluster The Cancer Genome Atlas (TCGA) EC samples into immune signature cluster modelling, evaluated immune cell profiling in UCEC cohort (n = 538) and defined four immune subtypes of EC. Next, we analysed the correlation between immune subtypes and clinical data including patient prognosis. Furthermore, we analysed the expression of immunomodulators and DNA methylation modification. The profiles of immune infiltration in TCGA UCEC cohort showed significant difference among four immune subtypes of EC. Among each immune subtype, natural killer T cells (NKT), dendritic cells (DCs) and CD8^+^T cells were significantly associated with EC patients survival. Each immune subtype exhibited specific molecular classification, immune cell characterization and immunomodulators expression. Moreover, the expression immunomodulators were significantly related to DNA methylation level. In conclusion, the identification of immune subtypes in EC tissues could reveal unique immune microenvironments in EC and predict the prognosis of EC patients.

**Highlights**

- Identified four immune subtypes in EC.
- Immune subtypes differ by immune cells, immunomodulator expression and patient survival.
- Proposing a new prognostic immune‐status classification of EC.

## 1 INTRODUCTION

Cancer remains one of the leading diseases with high mortality worldwide. Genetic mechanisms are crucially implied in cancer initiation, progression and metastasis. The Cancer Genome Atlas (TCGA) have become valuable resources of genetic data on a wide variety of tumours in human.^1^, ^2^ TCGA provide systematic information on DNA mutation, methylation, RNA expression and other comprehensive datasets on primary cancer tissues.^3^

Recent studies have shown that immunosuppressive microenvironment, including but not limited to tumour‐infiltrating lymphocytes (TILs), regulatory T cells (Treg), tumour‐associated macrophages (TAMs) and myeloid‐derived suppressor cells (MDSCs), could predict worse outcomes in solid tumours such as melanoma, breast, lung, ovarian, bladder, prostate and renal cancer.^4^ However, complex interaction between host immunity and solid tumour remains to be completely explored. Characterization of immune microenvironment based on gene expression signatures, immune subtypes, immunomodulators (IMs) expression and epigenetic modification, and the repertoires of B cell receptor (BCR) and T cell receptor (TCR) provides a wealth of information on tumour development.^5^ Consequently, immunotherapy has been developed as alternative or complementary treatment strategy for cancer to traditional radiotherapy and chemotherapy. In particular, CTLA‐4, PD‐1 and PD‐L1 antibodies have shown good efficacy in cancer treatment.^6^ Up‐regulation of inhibitory immune checkpoints by tumour‐infiltrating immune cells by cancer cells leads to cancer progression from host immunosurveillance. Changes in DNA methylation pattern and enrichment of methylated histone marks in the promoter regions could be major contributors to the up‐regulation of immune checkpoints (ICs) in the tumour microenvironment.^7^

Endometrial cancer (EC) is a common cancer in the woman, with an estimated 61 880 new cases and 12 160 deaths in the United States in 2019.^8^ EC develops in about 142 000 women worldwide and estimated 42 000 women may die from EC each year.^9^ Different treatment methods such as surgery, radiotherapy, brachytherapy and chemotherapy are currently applied to EC treatment based on the estimation of recurrence risk. However, current risk assessment system based on clinical, histological, imaging, biological prognostic factors is insufficient to account for evolutionary and prognostic heterogeneity of EC.

Therefore, in this study we performed extensive analysis based on TCGA UCEC cohort (Uterine Corpus Endometrial Carcinoma Cohort). We applied GVSA analysis, xCell method and multivariate Cox proportional hazards regression analysis to develop immune‐based prognostic signature of EC for better immunotherapy strategy.

## 2 METHODS

### 2.1 Datasets

The datasets were mRNA‐seq data derived from TCGA UCEC cohort (n = 538) (TCGA Data Portal at: https://tcga‐data.nci.nih.gov/tcga/, CGHub at: https://cghub.ucsc.edu/), with some modifications.^10^ AML samples were removed.^11^ Gene expression data were expressed as RNA‐Seq by Expectation Maximization (RSEM) normalized to the upper quartile of total reads.^12^, ^13^ Genomic subtypes within tissue types and TCGA Pan‐Cancer Cluster of Clusters Assignments (COCA) subtypes were based on TCGA tissue types defined previously.^14^, ^15^

### 2.2 Immune signature clustering

All EC samples (n = 538) available in TCGA were scored for 83 identified gene expression signatures. Next, GVSA enrichment analysis of all samples was performed.^16^ The published tumour immune expression signatures were compiled and scored across all non‐haematologic TCGA cancer types. Five immune signatures were then developed by unsupervised hierarchical clustering of mRNA‐seq expression data for 538 EC samples.^17^ In addition, other tumour‐infiltrating lymphocyte and macrophage gene signatures were obtained from published studies as follows: B cells (B cells naive, B cells memory), plasma cells, T cells CD8, T cells CD4 naive, T cells CD4 memory resting, T cells CD4 memory activated, T cells follicular helper, T cells regulatory (Tregs), T cells gamma delta, T helper cells (Th1, Th2), NK cells resting, NK cells activated, Monocytes, Macrophages M1 Macrophages M2, Dendritic cells resting, Dendritic cells activated, Mast cells resting, Mast cells activated, Eosinophils, and Neutrophils. The characteristic immune‐oncologic gene signatures were then used to cluster TCGA EC samples into four subtypes and were defined by immunomodulator (IM) gene analysis. Survival modelling was performed to assess the association of immune subtypes with patient prognosis. TCR and BCR repertoire inference and immunomodulator expression and regulation were characterized in the context of TCGA‐defined molecular subtypes, and these four immune subtypes, so as to assess the relationship between factors affecting immunogenicity and immune infiltrate.

### 2.3 IM gene expression correlation with dna methylation

To study the relationship between gene expression and DNA methylation of immunomodulators, we mapped DNA methylation probes to genes using bioconductor packages *IlluminaHumanMethylation450kanno.ilmn12.hg19* containing manifests and annotation for Illumina's 450 k arrays. From *IlluminaHumanMethylation450kanno.ilmn12.hg19*, 620 loci were identified. For a given IM gene, Spearman's correlation between gene expression and each corresponding gene‐associated probe was evaluated, within each immune subtype. Results were then filtered to retain sets of probes with similarly signed correlations, to reduce noise and increase robustness of signal. The filter produces probe‐clusters, where probes are uniquely assigned a cluster, are within 10 KB and have the same correlation sign. Single correlation values per probe‐cluster were found by averaging probes.

### 2.4 Statistical analysis

Comparison was analysed by one‐way analysis of variance (ANOVA). Correlation was analysed by Pearson's method. Univariate analysis was performed by using Cox proportional hazards model with each signature as a continuous variable. Prognostic value of individual gene expression signature was assessed by using multivariable Cox proportional hazards model. All survival analyses were performed as described previously.^18^ Two‐sided *P* < 0.05 was considered significant.

## 3 RESULTS

### 3.1 EC immune subtypes

**Figure 1:**
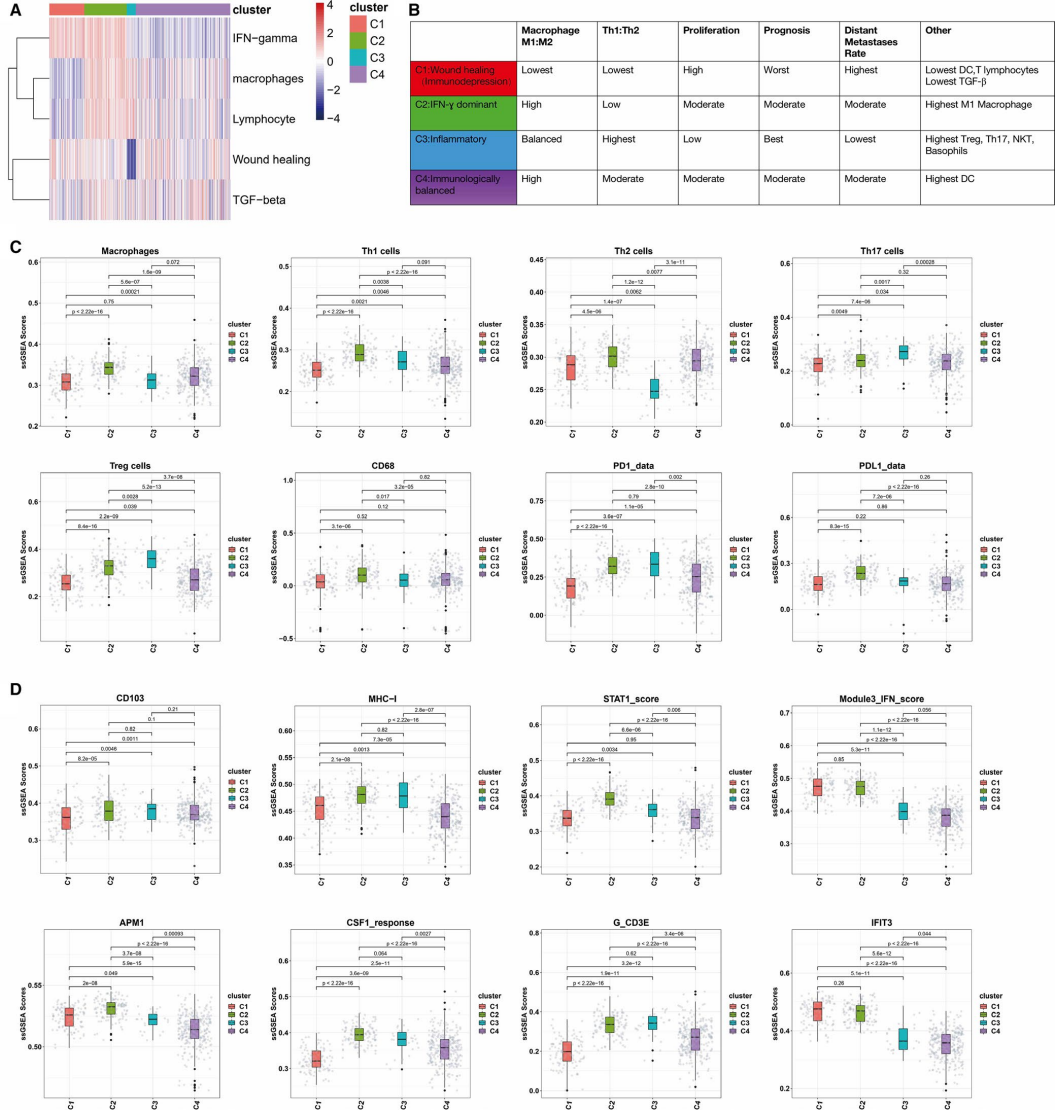
Immune subtypes of EC. A, Expression signature modules. Representative gene expression signatures for each module (columns) to group TGCA EC samples (rows), and four immune subtypes C1‐C4 were identified. Five modules of shared associations are indicated by boxes. B, Key characteristics of immune subtypes, including immune cells and IM gene expression. C, The proportion of major immune cells for different immune subtypes. D, The proportion of major IM gene expression for different immune subtypes

First, we performed GSVA analysis to identify immune cell profiles in EC based on TCGA UCEC cohort. Cluster analysis of known 83 immune expression signatures revealed five immune expression signatures in EC, including macrophages/monocytes,^19^ lymphocyte infiltration (dominated by T, B cells),^20^ TGF‐β response,^21^ IFN‐ɣ response^22^ and wound healing.^23^ These signatures were then used to cluster TCGA EC types into four immune subtypes C1‐C4 (Figure 1A).

C1 (immunodepression) was wound healing and IFN‐ɣ dominant, had the lowest Th1:Th2 ratio and the lowest expression of PD1and PDL1, and had the poorest immunologic activity. C2 (IFN‐ɣ dominant type) had higher IFN‐ɣ, lymphocyte and macrophage. C3 (inflammatory type) had high lymphocyte and macrophage, the lowest wound healing and the highest expression of PD1, and had the highest Th17 cells and Treg cells, the highest Th1/Th2 cell proportion and the strongest immunologic activity. C4 (immunologically balanced type) had a relatively balanced distribution of those cells, reflecting a state of balanced immune condition (Figure 1B). Each cancer immune subtype exhibited a subtype‐specific immune cell characterization (Figure 1C). In addition, each cancer immune subtype exhibited a subtype‐specific immune molecule expression characterization (Figure 1D). For example, the relative levels of the interferon (IFN)‐inducible gene‐IFIT3 and IFN were elevated in C1 but were significantly lower in C3.

### 3.2 Composition of tumour immune infiltrates

**Figure 2:**
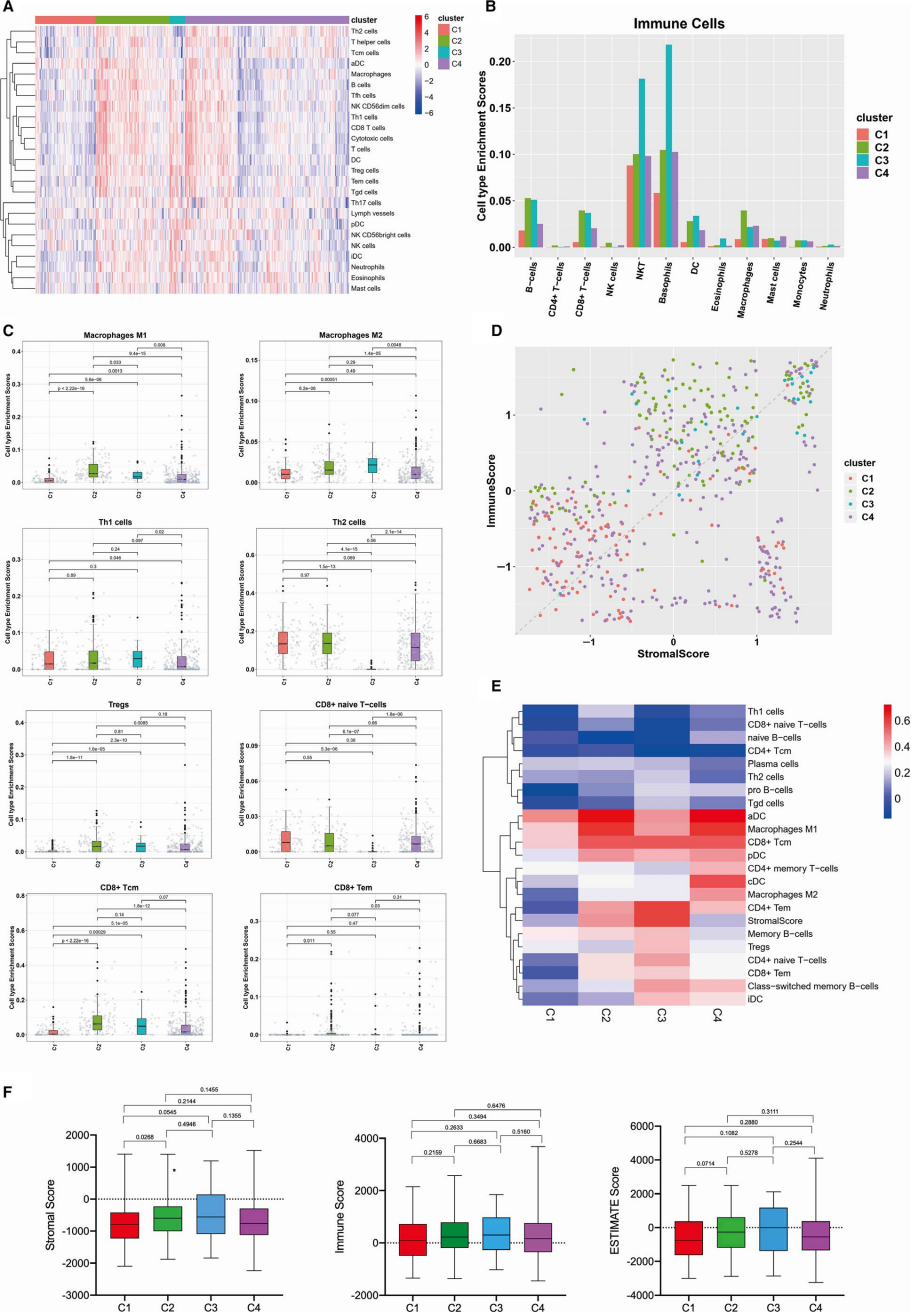
Composition of tumour immune infiltrate in EC. A, The heatmap of immune cells for different immune subtypes. For each immune subtypes, we calculated the median of the absolute score of the 22 cell types given by the CIBERSORT in each cohort. B, The proportion of major classes of immune cells (from CIBERSORT) for different immune subtypes. C, List of major immune cell distribution in different EC immune subtypes. D, Immune score (*y* axis) versus stromal score in the TME (*x* axis) for four representative immune subtypes. Dots represent individual tumour samples. E, The correlation between Immune Score's Spearman and the heatmap in each group. F, Immune score, stromal score and calculated ESTIMATE score in different immune subtypes. Box plot shows that there is significant association between immune subtypes and the level of above mentioned scores

The heatmap of immune cells for 4 different immune subtypes is shown in Figure 2A. We found that different immune cells showed significant changes among EC with 4 different immune subtypes. In particular, the numbers of NKT and basophilic were significantly higher in C3 (Figure 2B). The detailed comparisons of major immune cells distribution in different EC immune subtypes are shown in Figure 2C.

Estimation of STromal and Immune cells in MAlignant Tumours using Expression data (ESTIMATE) is a concept based on gene expression signatures to evaluate stromal and immune cells in tumour samples.^24^ Therefore, we first analysed the correlation of stromal scores and immune scores which indicated the levels of infiltrating stromal and immune cells, respectively, in EC immune subtypes (Figure 2D and Figure S1). Next, we analysed the correlation between immune score's Spearman and the heatmap in each EC immune subtype. Macrophages M1, pDC, aDC, CD8^+^ Tcm, CD4^+^ memory T cells were more relevant to immune scoring. (Figure 2E). Finally, we calculated stromal, immune and ESTIMATE scores to judge tumour purity in EC tissues. The results showed that the scores had no significant differences except stromal scores in C1 immune subtype (Figure 2F).

### 3.3 Prognostic significance of immune subtypes

**Figure 3:**
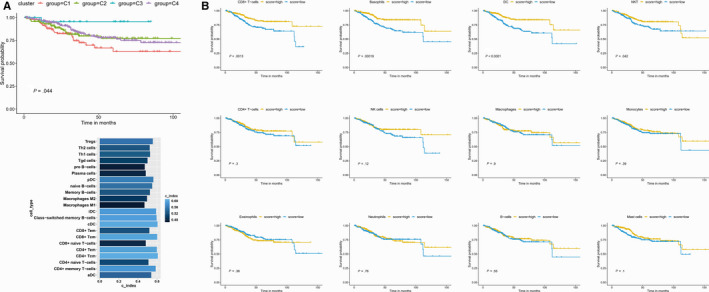
Prognostic significance of EC immune subtypes. A, OS of EC patients by based on immune subtypes. B, The K‐M survival analysis of OS of major immune cells in TCGA UCEC cohort, including B cells, T cells CD8, T cells CD4, Tregs, Th1, Th2, NK cells, macrophages, dendritic cells, mast cells resting, eosinophils and neutrophils. C, Cox regression analysis of C‐index value of different immune cell types. A higher c‐index value indicated a greater impact on prognosis

Next, we wondered whether different immune subtypes could significantly distinguish overall survival of EC patients. We found that C3 had the best prognosis, whereas C2 and C4 had less favourable outcomes although they contained substantial immune component, and C1 had the least favourable outcome (Figure 3A). Moreover, Kaplan‐Meier curve analysis of immune subtypes showed that natural killer T cells (NKT), dendritic cells (DCs), CD8^+^T cells, basophils were significantly associated with OS in EC patients (Figure 3B). To confirm prognostic significance of different immune subtypes, we calculated concordance index (C‐index).^25^ The immune cell types were the most significant prognostic variables, with the highest C‐index in CD4^+^Tcm, cDC, CD4^+^Tem (Figure 3C).

### 3.4 Key immune cells differed in different stages of EC

**Figure 4:**
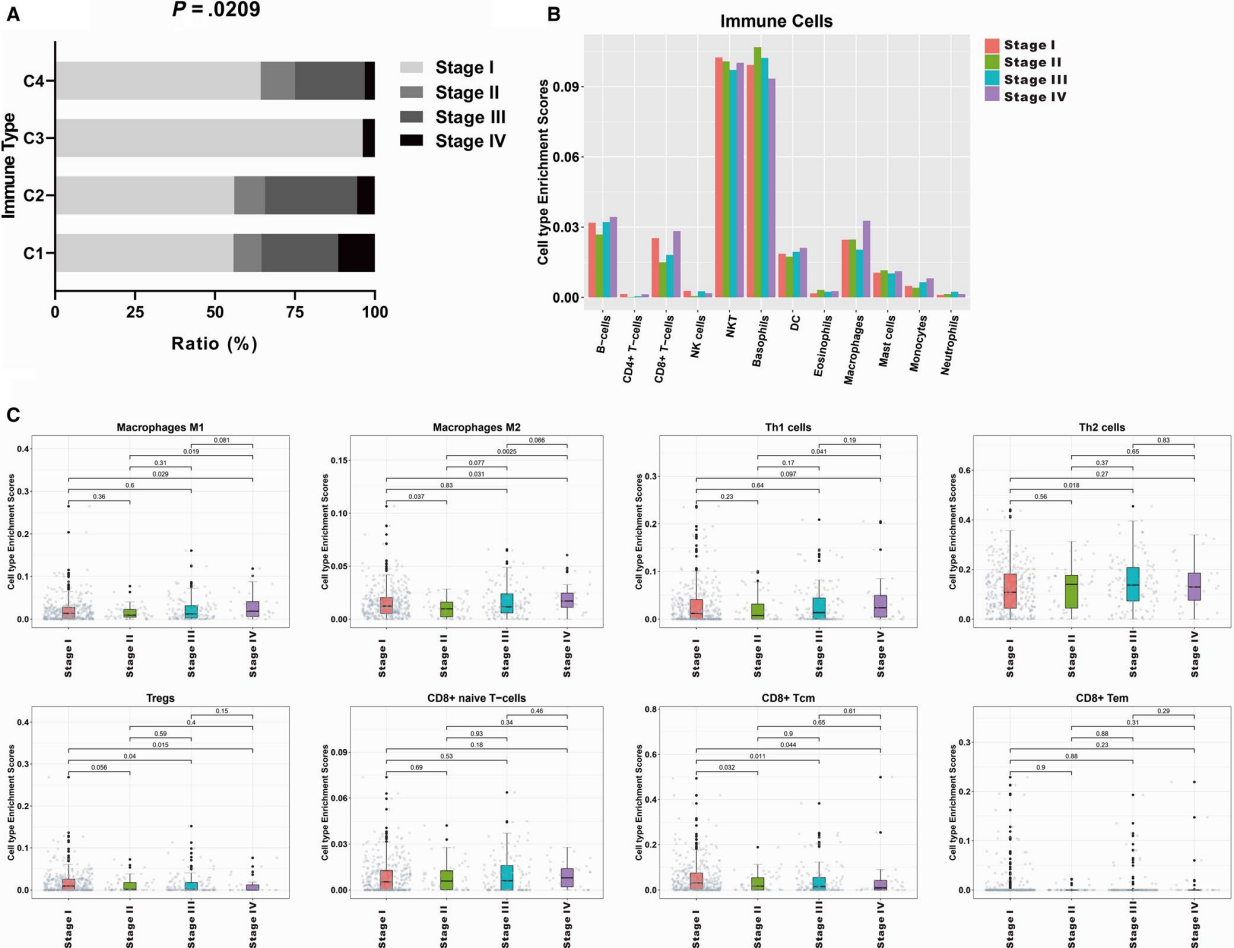
Values of key immune cells differed in different EC tumour stage. A, The proportion of different immune subtypes in different EC tumour stage. B, The proportion of major immune cells in different EC tumour stage. C, List of major immune cell distribution in different EC tumour stage

We also wondered whether different immune subtypes were associated with stage of EC. The results showed the proportion of different immune subtypes in different EC tumour stage. Although C1 and C2 showed similar distribution of EC tumour stage, C3 and C4 showed significantly different distribution of EC tumour stage (Figure 4A). The proportion of major immune cells was also different in different EC tumour stage (Figure 4B). In particular, macrophages M1, macrophages M2, Th1, Th2, Tregs, CD8^+^T naive cells, CD8^+^ Tcm, and CD8^+^ Tem cells showed significant changes among EC with different stages (Figure 4C and Figure S2).

### 3.5 Immunomodulators expression in EC samples

**Figure 5:**
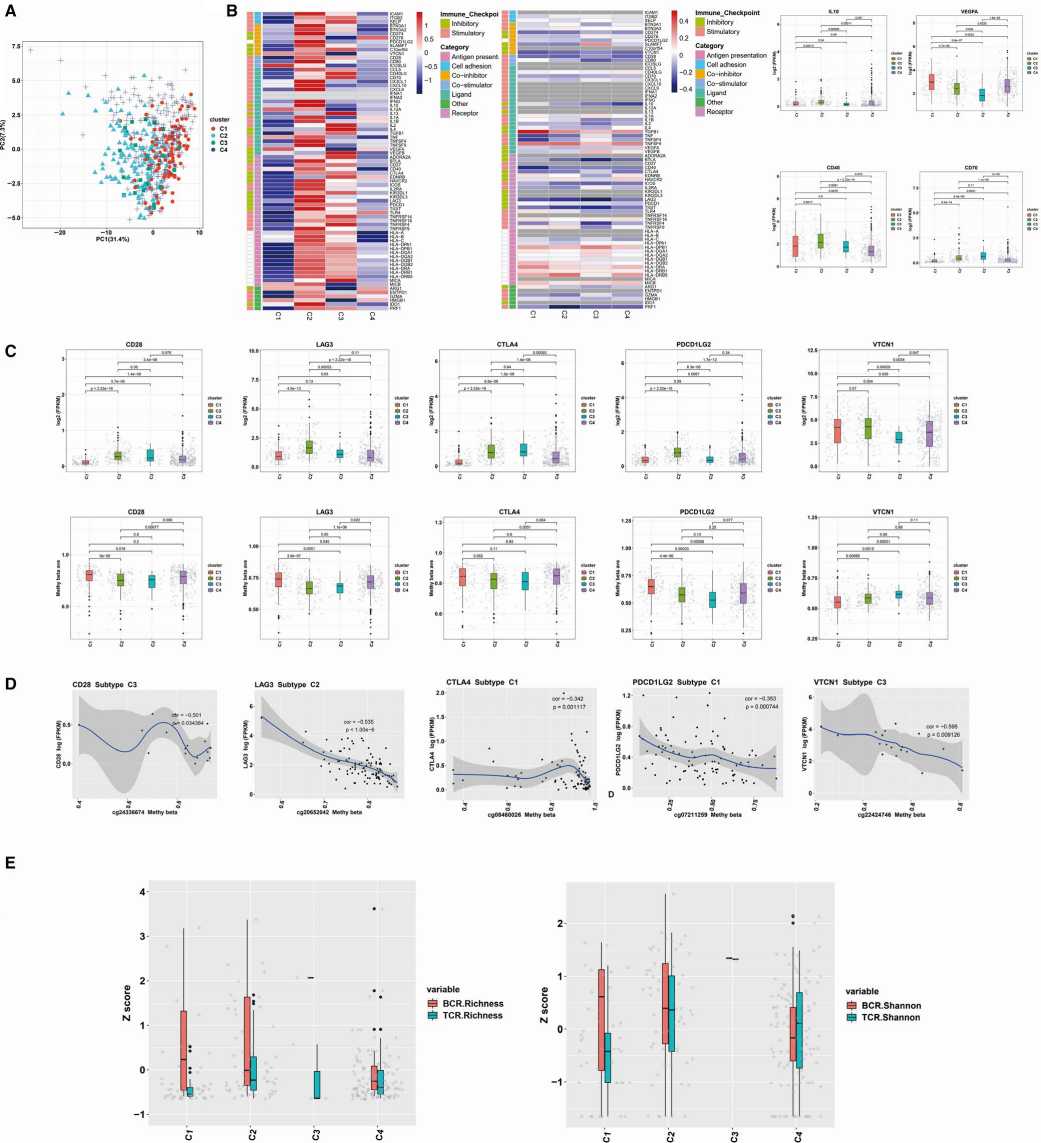
Regulation of immunomodulators and IM genes Expression Correlation with DNA Methylation. A, Principal component analysis between four immune subtypes based on immune genes. Dots represent individual tumour samples. B, Distribution of log‐transformed expression levels for IM genes with the largest differences across subtypes by Kruskal‐Wallis test. C, CD28, LAG3, CTLA4, PDL2 and VCTN1 expression were inversely correlated to methylation levels in certain immune subtypes. Each point represents a tumour sample, and colour indicates point density. D, From left to right: mRNA expression (median normalized expression levels); expression versus methylation (gene expression correlation with DNA methylation beta‐value); the differences in IM genes in the particular subtype and the amplification fraction in all samples); and the deletion frequency (as amplifications) for 75 IM genes by immune subtypes. E, BCR and TCR diversity measured by species richness and Shannon entropy in different immune subtypes

Since agonists and antagonists of immunomodulators (IMs) are increasingly used in cancer immunotherapy,^26^ we retrieved immunomodulatory genes from TCGA and 78 IMs were listed in Table S1. Next, we conducted principal component analysis (PCA) to investigate the difference between four immune subtypes based on immune genes expression profiles. The results showed that the four groups were generally distributed in different directions (Figure 5A).

Kruskal‐Wallis test showed that gene expression of IMs varied across EC immune subtypes, and genes with the greatest differences between subtypes included IL10, VGEF‐α, CD40 and CD70 (Figure 5B). Seventy‐five genes were implicated as possible regulators of IM gene expression; among them, several genes associated with IMs in multiple subtypes included immune inhibitors (VTCN1, LAG3, PDL2 and CTLA4) and activators (CD28) (Figure 5C).

Furthermore, we examined the relationship between IM gene expression and DNA methylation. We extracted all IM gene methylation information from 583 methylation profiled UCEC cohort samples were downloaded in the form of raw files for the 450 k array from the TCGA data and focused on the differences in the methylation of the IM genes in different immune subtypes. The results showed that DNA methylation of many IM genes, for example CD28, LAG3, CTLA4, PDL2 and VTCN1, was inversely correlated with gene expression (Figure 5D).

In addition, B cell receptor (BCR) and T cell receptor (TCR) repertoire analysis showed that BCR and TCR diversity measured by both species richness and Shannon entropy exhibited significant differences in four immune subtypes (Figure 5E).

## 4 DISCUSSION

Although most EC patients can be diagnosed and treated at the early stage, 15% patients are diagnosed at the locally advanced or occult metastatic stage and suffer from tumour recurrence due to limited response to surgery and radiotherapy.^9^ In addition, the traditional dualistic model classification theory on EC, which is based on the presence or absence of oestrogen nuclear receptor expression, has limited significance in prognostic judgment and therapeutic guidance for patients, suggesting the highly heterogeneous clinical and biological characteristics of EC.^27^, ^28^ Recently, molecular classification theory based on sequencing analysis has compensated for the limitations of traditional classification to some extent, which categorizes EC into four types, including POLE hypermutation, microsatellite instability, endometrial‐like low copy number and serous high copy number. Nonetheless, apart from POLE hypermutation, other common gene mutations in EC could not be accurately classified according to the above molecular classification. For example, the most common PTEN and PIK3CA gene mutations in EC are distributed in EC patients with all the four molecular types.^29^ Therefore, it is urgent to develop better EC classification system for clinical intervention of EC from the perspective of overall characteristics of tumour microenvironment (TME).

TME composed of tumour cells, stromal cells and immune cells has been considered as the ‘fertile soil’ for malignant transformation of tumour.^30^ Cancer‐associated fibroblasts (CAFs) in the microenvironment promote EC metastasis through down‐regulating the expression of miR‐148b and other molecules in EC cells. Among them, the immune suppressive microenvironment plays a decisive role in the ‘unbridled’ survival and progression of tumour cells, which is also related to the immune escape capacity of tumour cells.^31^, ^32^ A recent study summarized the immune characteristics of multiple tumours, including the immune subtypes of six tumour types, and showed that the tumour immunotyping is closely associated with patient prognosis.^33^ However, systematically investigation in immune characteristics of EC is not completely understood. Moreover, it remains to be further clarified about the differences in tumour immune characteristics among EC patients, as well as the role of immune microenvironment status in patient prognosis.

In this study, we assessed all EC samples for immunological aspects using multiple methods, such as the estimate of immune cell fractions based on gene expression and DNA methylation data, and the evaluation of BCR and TCR repertoires based on RNA sequencing data. Immunological aspects were compared among EC tissues with different immune subtypes and stages, and analysed for the correlation with the changes in TME and EC patient outcome.

Based on high‐throughput transcriptome and methylation sequencing data from 538 EC cases collected from TCGA, we stratified EC into four immune subtypes: C1 (wound healing type), which had the worst immune activity and the least infiltration of macrophages and lymphocytes; C2 (IFN‐γ dominant type), in which IFN‐γ pathway was activated, and macrophages were polarized to M1 type; C3 (inflammatory type), which had the strongest immune activity and the most infiltration of macrophages and lymphocytes; and C4 (immunologically balanced type), which had balanced infiltration of immune cells and activation of immune pathways. More importantly, we found that EC patients in C1 had the highest metastatic rate with the worst overall prognosis, whereas those in C3 had the highest overall survival rate. These results indicate that the immune characteristics of TME can serve as a new basis for EC classification, which are closely associated with patient prognosis.

Immune cell infiltration has become a new indicator of prognosis in patients with different types of solid tumours.^34^ In this study, we divided EC samples into four immune subtypes C1‐C4 based on immune and stromal cell infiltration into EC tissues. C1 subtype indicated the worst prognosis and exhibited the composite signature with dominant IFN‐ɣ and wound healing and low macrophage infiltration, in agreement with immunosuppressed TME which would favour a poor outcome. In contrast, C3 subtype exhibited type I immune response with high Th1/Th2 ratio and indicated the favourable prognosis, in agreement with previous opinion that a dominant type I immune response would inhibit tumorigenesis.^35^ Furthermore, C3 subtype exhibited pronounced Th17 signature, consistent with recent data that Th17 signature is correlated with better survival of cancer patients.^36^ In contrast, C2 subtype was IFN‐ɣ dominant and indicated less favourable survival, although it exhibited CD8 T cell signature with high M1 Macrophage content, which would support strong anti‐tumour immunity.^37^ Human tumour‐associated macrophages (TAMs) could be isolated from primary breast, lung, colorectal, and endometrial cancers exhibited a similar capability in invasion and metastasis; meanwhile, in our research, TAMs were found to be in infiltrated the most in stage IV patients. As development and progression of cancer, such as colorectal cancer (CRC), are known to be affected by the immune system, cell subsets such as T cells, natural killer (NK) cells and NKT cells are considered interesting targets for immunotherapy and clinical biomarker research. Those patients with a high percentage of inhibitory receptor circulating NKT‐like cells showed a trend towards shorter DFS.^38^ As in endometrial cancer, recent research proved it that the tumour microenvironment reshapes NK cell phenotype and leads to promote tumour progression.^39^ The balance between Th1 and Th2 is important for the immune system homeostasis.^40^ TGF‐β may affect Th1‐Th2 balance within the tumour microenvironment. Collectively, our results suggested that immune cell profiles in TME of EC tissues could predict prognosis outcome of EC patients.

It is known that abnormal DNA methylation is implicated in tumorigenesis, since it inhibits the expression of key genes involved in regulating cancer cell differentiation, proliferation and invasion.^41^ The extent of immune cell infiltration into solid tumours are key determinants of therapeutic response. Some studies demonstrated that both DNA hypomethylation and H3K9me3 and H3K27me3 repressive histones get involved in up‐regulation of certain IM genes, such as CTLA‐4 and TIGIT. However, repressive histones modifications, but not DNA hypomethylation, are involved in up‐regulation of PD‐1 and TIM‐3 genes in CRC tumour tissue. Due to DNA methylation and histone modification often have cross‐talk in gene expression, our results suggested that the expression profile of those critical IM genes in TME of EC tissues may be induced by DNA methylation.^42,43^ Our previous study showed that EC‐related DNA hypermethylation accelerated malignant progression of EC.^44^ In this study, we detected IMs gene DNA methylation status in EC tissues of different immune subtypes and found that DNA methylation of many IM genes showed negative correlation with their expression, which links crucial IM gene expression with DNA methylation patterns in TME of EC and supplies a better understanding of how DNA methylation acting on specific immune‐cellular tumour progression pathways contribute to prognostic stratification. Further characterization of DNA methylome in immune microenvironment of EC will help elucidate the mechanism of cancer resistance to immunotherapy, enable customized treatment design and develop novel diagnostic, therapeutic and prognostic markers of EC.

**Figure 6:**
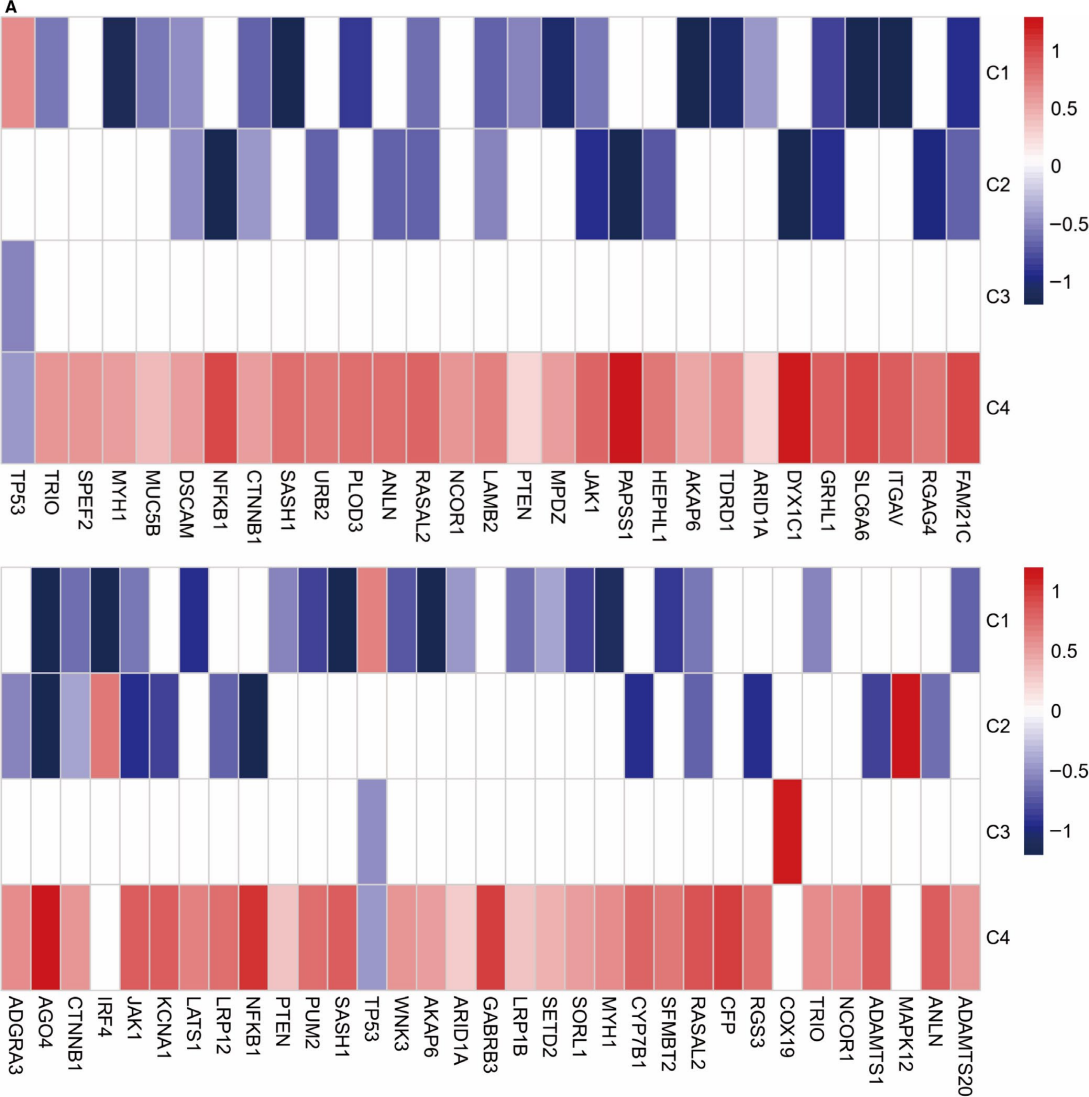
Mutations in driver genes and oncogenic mutations (OM) within immune subtypes. A, Enrichment and depletion of mutations in driver genes and oncogenic mutations (OM) within immune subtypes, displayed as fold enrichment (white, no significant relative association)

The caveat to the use of TCGA data should be noted. We also correlated mutations in almost 300 cancer driver genes with immune subtypes and found 30 significant associations (Figure 6). C1 was enriched in mutations in driver gene‐TP53. C4 was the most enriched in mutations in driver gene, such as NF‐ĸB, PTEN, ARID1A. So those driver mutations differing from different immune subtypes seem to have no correlations within immune subtypes. However, driver mutations such as TP53, by inducing genomic instability, may alter the immune landscape via the generation of neoantigens and then cause tumour progression. Our results are limited by the restriction to data based on genome‐wide analysis. Therefore, we could not evaluate complex immune cell phenotypes and in TME in EC tissues. Further work is needed to determine the functional aspects of these associations, which will be important to verify our conclusion.

In conclusion, we identified four immune subtypes in EC tissues. The specific features of these immune subtypes were associated with gene expression signatures and epigenetic modulation of immunoregulatory genes and immune cells, which could shape unique immune microenvironments in EC and predict the prognosis of EC patients.

## CONFLICT OF INTEREST

The authors declared no competing interest.

## AUTHOR CONTRIBUTION

LBL performed the research, LBL analysed the data and wrote the paper, WXP designed the research study and wrote the paper.

## Supporting information

Fig S1

Fig S2

Table S1
