# Supplementary figures and images for: Prognostic significance of immune landscape in tumour microenvironment of endometrial cancer
Source: J Cell Mol Med. 2020 May 19;24(14):7767–77. doi: 10.1111/jcmm.15408 (PMC7348174; doi:10.1111/jcmm.15408)

A

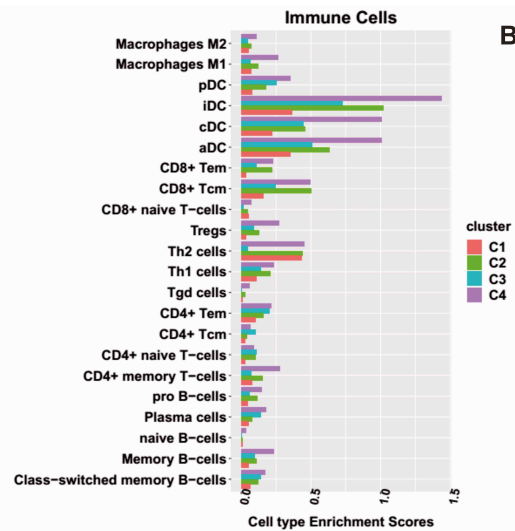

B

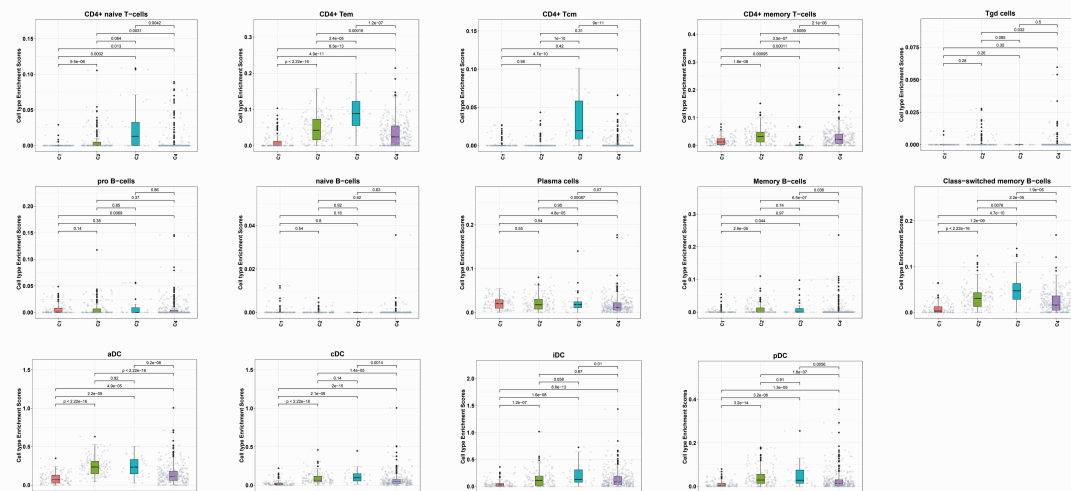

C

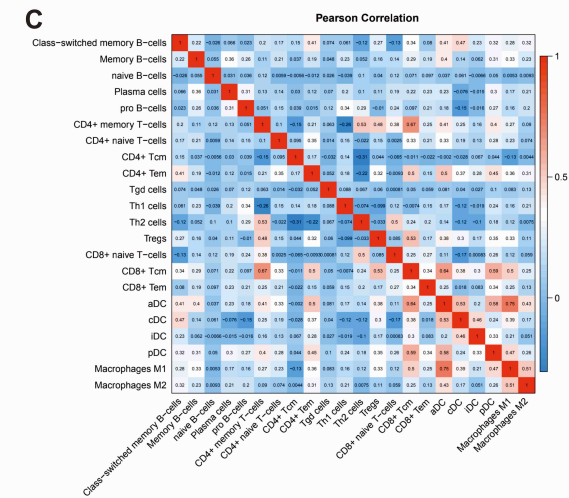

Supplement: Supplementary file 1 — Fig S1 [file JCMM-24-7767-s001.pdf]

A

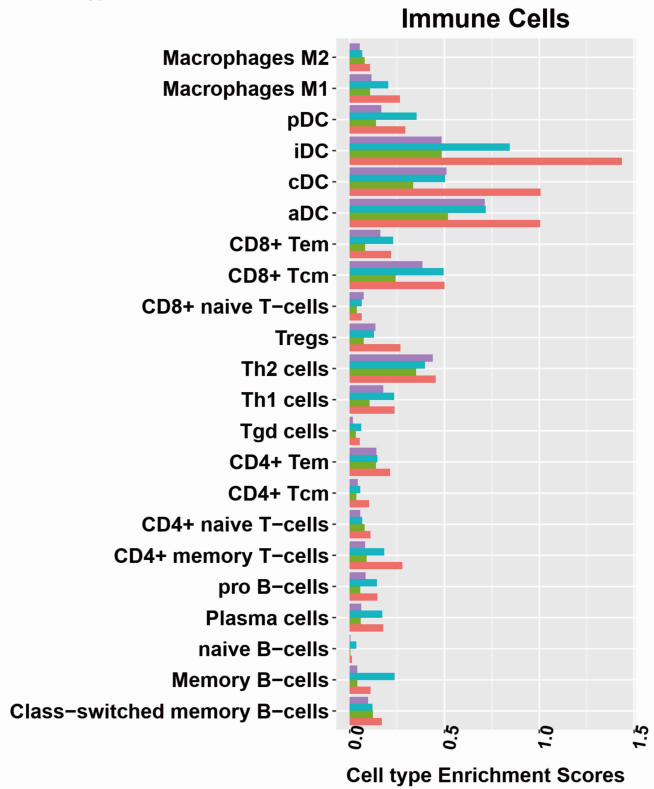

B

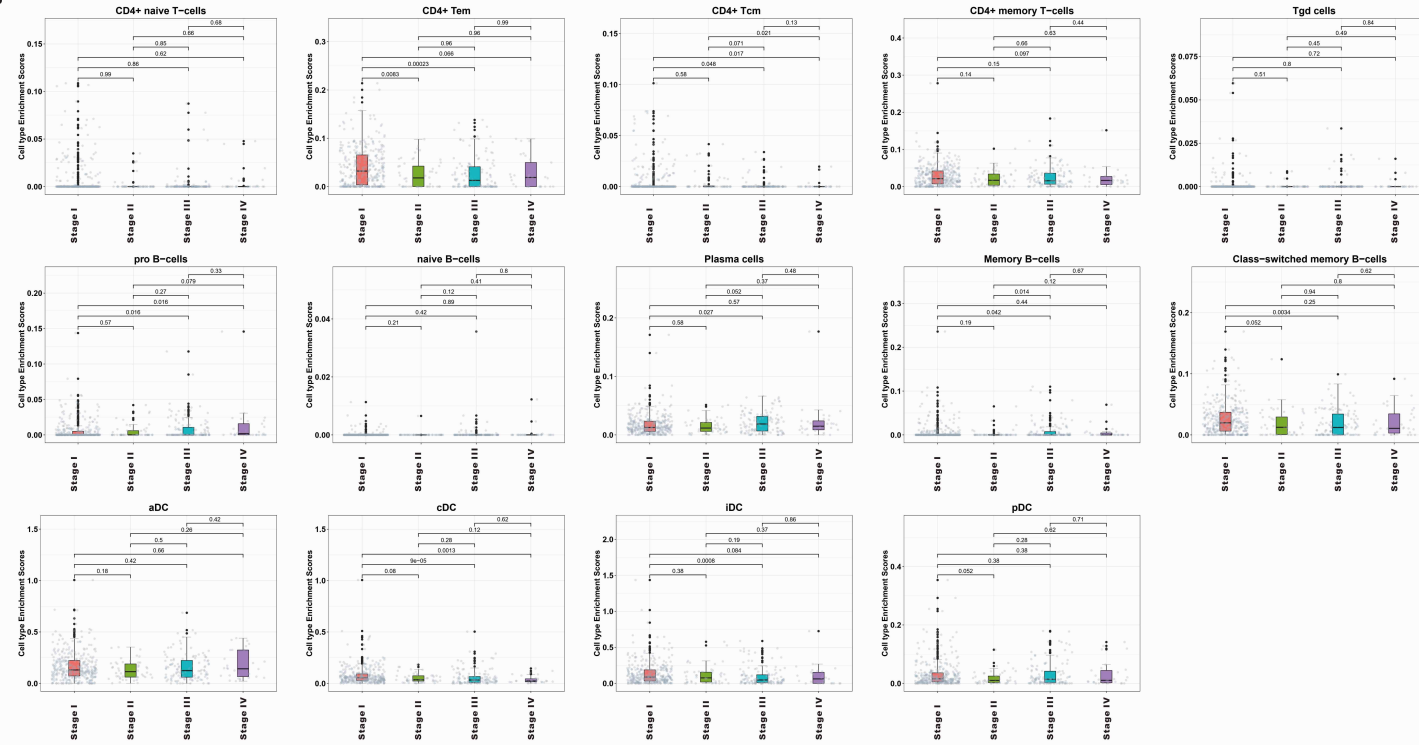

Supplement: Supplementary file 2 — Fig S2 [file JCMM-24-7767-s002.pdf]
