# Supplementary material for: Prognostic significance of immune landscape in tumour microenvironment of endometrial cancer
Source: J Cell Mol Med. 2020 May 19;24(14):7767–77. doi: 10.1111/jcmm.15408 (PMC7348174; doi:10.1111/jcmm.15408)
Supplement: Supplementary file 3 — Table S1 [file JCMM-24-7767-s003.docx]

| gene | P.value | FDR |
| --- | --- | --- |
| ICAM1 | 8.55E-13 | 2.65E-11 |
| ITGB2 | 6.61E-33 | 4.96E-31 |
| SELP | 4.84E-14 | 1.69E-12 |
| BTN3A1 | 6.53E-13 | 2.15E-11 |
| BTN3A2 | 3.84E-15 | 1.46E-13 |
| CD274 | 1.21E-19 | 6.19E-18 |
| CD276 | 0.13462 | 0.673101 |
| PDCD1LG2 | 5.81E-17 | 2.44E-15 |
| SLAMF7 | 2.23E-27 | 1.56E-25 |
| C10orf54 | 1.32E-21 | 8.07E-20 |
| VTCN1 | 0.000476 | 0.006659 |
| CD28 | 2.71E-20 | 1.48E-18 |
| CD80 | 1.15E-10 | 3.32E-09 |
| ICOSLG | 0.006096 | 0.067056 |
| CCL5 | 1.21E-26 | 8.19E-25 |
| CD40LG | 4.77E-23 | 3.05E-21 |
| CD70 | 1.16E-16 | 4.77E-15 |
| CX3CL1 | 0.001501 | 0.018011 |
| CXCL10 | 5.17E-26 | 3.46E-24 |
| CXCL9 | 2.22E-20 | 1.24E-18 |
| IFNA1 | 8.49E-05 | 0.001529 |
| IFNA2 | 0.080592 | 0.483552 |
| IFNG | 5.04E-17 | 2.17E-15 |
| IL10 | 2.71E-05 | 0.000596 |
| IL12A | 6.81E-05 | 0.001294 |
| IL13 | 0.024852 | 0.198816 |
| IL1A | 0.036562 | 0.255934 |
| IL1B | 3.79E-12 | 1.14E-10 |
| IL2 | 0.000405 | 0.006072 |
| IL4 | 0.143812 | 0.673101 |
| TGFB1 | 1.66E-07 | 4.33E-06 |
| TNF | 5.32E-05 | 0.001064 |
| TNFSF4 | 0.012124 | 0.109113 |
| TNFSF9 | 0.000174 | 0.002791 |
| VEGFA | 6.87E-09 | 1.86E-07 |
| VEGFB | 0.325284 | 0.975851 |
| ADORA2A | 0.000874 | 0.011357 |
| BTLA | 1.16E-18 | 5.58E-17 |
| CD27 | 9.35E-32 | 6.92E-30 |
| CD40 | 1.27E-14 | 4.69E-13 |
| CTLA4 | 2.44E-21 | 1.41E-19 |
| EDNRB | 0.01024 | 0.1024 |
| HAVCR2 | 1.13E-27 | 8.11E-26 |
| ICOS | 1.30E-21 | 8.07E-20 |
| IL2RA | 3.98E-20 | 2.07E-18 |
| KIR2DL1 | 8.94E-06 | 0.000206 |
| KIR2DL3 | 0.000116 | 0.00198 |
| LAG3 | 2.28E-17 | 1.00E-15 |
| PDCD1 | 1.81E-19 | 9.07E-18 |
| TIGIT | 8.06E-24 | 5.24E-22 |
| TLR4 | 4.42E-18 | 1.99E-16 |
| TNFRSF14 | 2.71E-13 | 9.21E-12 |
| TNFRSF18 | 4.67E-05 | 0.00098 |
| TNFRSF4 | 2.83E-16 | 1.13E-14 |
| TNFRSF9 | 2.12E-20 | 1.21E-18 |
| HLA-A | 2.25E-21 | 1.33E-19 |
| HLA-B | 1.61E-27 | 1.15E-25 |
| HLA-C | 3.11E-20 | 1.65E-18 |
| HLA-DPA1 | 8.21E-26 | 5.42E-24 |
| HLA-DPB1 | 1.00E-26 | 6.93E-25 |
| HLA-DQA1 | 5.53E-30 | 4.03E-28 |
| HLA-DQA2 | 2.03E-10 | 5.68E-09 |
| HLA-DQB1 | 2.69E-20 | 1.48E-18 |
| HLA-DQB2 | 8.21E-13 | 2.63E-11 |
| HLA-DRA | 1.54E-21 | 9.21E-20 |
| HLA-DRB1 | 1.10E-22 | 6.92E-21 |
| HLA-DRB5 | 2.44E-19 | 1.20E-17 |
| MICA | 0.534393 | 1 |
| MICB | 5.21E-06 | 0.000125 |
| ARG1 | 0.516447 | 1 |
| ENTPD1 | 4.15E-07 | 1.04E-05 |
| GZMA | 1.17E-18 | 5.58E-17 |
| HMGB1 | 1.45E-15 | 5.67E-14 |
| IDO1 | 2.51E-14 | 9.03E-13 |
| PRF1 | 2.05E-18 | 9.42E-17 |
